# Process Optimization and Tailored Mechanical Properties of a Nuclear Zr-4 Alloy Fabricated via Laser Powder Bed Fusion

**DOI:** 10.3390/mi14030556

**Published:** 2023-02-27

**Authors:** Changhui Song, Zhuang Zou, Zhongwei Yan, Feng Liu, Yongqiang Yang, Ming Yan, Changjun Han

**Affiliations:** 1 School of Mechanical and Automotive Engineering, South China University of Technology, Guangzhou 510641, China; 2 Institute of Reactor Waste and Radiochemistry Research, China Nuclear Power Technology Research Institute Co., Ltd., Shenzhen 518028, China; 3 Department of Materials Science and Engineering, Southern University of Science and Technology, Shenzhen 518055, China

**Keywords:** additive manufacturing, laser powder bed fusion, zirconium alloys, Zr-4, process optimization

## Abstract

A nuclear Zr-4 alloy with a near full density was fabricated via laser powder bed fusion (LPBF). The influences of process parameters on the printability, surface roughness, and mechanical properties of the LPBF-printed Zr-4 alloy were investigated. The results showed that the relative density of the Zr-4 alloy samples was greater than 99.3% with the laser power range of 120–160 W and the scanning speed range of 600–1000 mm/s. Under a moderate laser power in the range of 120–140 W, the printed Zr-4 alloy possessed excellent surface molding quality with a surface roughness less than 10 µm. The microstructure of the printed Zr-4 alloy was an acicular α phase with an average grain size of about 1 µm. The Zr-4 alloy printed with a laser power of 130 W and a scanning speed of 400 mm/s exhibited the highest compression strength of 1980 MPa and the highest compression strain of 28%. The findings demonstrate the potential in the fabrication of complex Zr-4 alloy parts by LPBF for industrial applications.

## 1. Introduction

Zirconium and its alloys have been indispensable materials for the development of the atomic energy industry due to their small thermal neutron-capturing cross-sections and outstanding nuclear properties [1]. In particular, Zr-4 alloys, the key materials of nuclear reactors, possess excellent nuclear properties, mechanical properties, corrosion resistance, and thermal stability in high-temperature environments, which can be used in fuel envelope positioning lattices of pressurized water reactors, component boxes, and heat exchangers [2,3]. Zr alloys often consist of a single α phase at room temperature. The α phase can be obtained from the transformation of a β phase, when the phase transition temperature is lower than 865 °C. In addition, non-equilibrium phase transitions (such as martensitic phase transition) may occur in Zr alloys to form α’/α’’ phases [4].

Traditional manufacturing efforts have been made on Zr alloys fabricated by casting. Fuloria et al. [5] studied the mechanical properties and microstructure changes of a multi-axial forged Zr-4 alloy under different cumulative strains at low temperatures. The results showed that the ultimate tensile strength and hardness of 5.91 cumulative strain increased from 474 MPa to 717 MPa and from 190 HV to 238 HV, respectively. Such increases in strength and hardness are influenced by the grain size effect and high dislocation density. Li [6] investigated the microstructure evolution of a rolled Zr-4 alloy followed by annealing. After annealing at 480−580 °C for 2 h, the grain boundary morphology of the Zr-4 alloy evolved from relatively blurred to equiaxial. Qiu [7] identified the second phase in the Zr-4 alloy as a Zr_x_(Fe_2_Cr) compound, which was distributed between the dendrites of the α-Zr matrix.

Laser additive manufacturing has been used for fabricating Zr alloys, which overcomes challenges faced by traditional processes to manufacture fully dense metal parts with complex geometries and good mechanical properties [8]. Su [9] studied the formation of a Zr-based bulk metallic glass by laser-directed energy deposition additive manufacturing. A crack-free Zr-based bulk metallic glass was successfully fabricated with a sufficient high ultimate tensile strength of 880 MPa, as well as a high yield strength of 835 MPa, which was similar to that of the as-cast alloy. 

Laser powder bed fusion (LPBF) is another laser additive manufacturing technology suitable for fabricating bulk parts from metal powders. LPBF uses a high-energy laser beam to melt metal powders [8]. First, a thin layer of metal powder is laid on a substrate. Then, the laser scans and melts the powder layer through a predefined path. The powder layers are continuously melted layer by layer, resulting in accumulation to obtain the geometrically complex parts with a high accuracy [10,11]. Sun [12] focused on the effect of annealing on the mechanical properties of a Zr-1Mo alloy fabricated by LPBF. The ultimate tensile strength and elongation of the alloy were 964 MPa and 11.0%, respectively, after annealing at 803 K for 2 h. A good balance of the strength and toughness was achieved, when α phase experienced stress relief. Currently, there has been limited research works on Zr-4 alloys fabricated by LPBF. 

During LPBF, the volumetric energy density is determined by the laser power, scanning speed, hatch spacing, and layer thickness, which has a great influence on the relative density of printed parts. Particularly, the laser power and scanning speed are the main factors affecting the energy density. The hatch spacing may affect the overlapping ratio of melt tracks. A large hatch could cause microholes and unmelted powder particles [13]. The layer thickness determines the interlayer combination, and an excessive layer thickness leads to the lack of fusion between layers and severe spattering [14]. Low energy densities are prone to resulting in unmelted powder particles due to insufficient energy input and balling phenomena [15]. Comparatively, high energy densities may lead to excessive recoil pressure and form deep melt pools. The gas from the powder or atmospheric gas inside the chamber is thus difficult to escape and trapped inside the melt pools to form gas pores [16]. Additionally, high energy densities may lead to stress cracking that results from large residual stress caused by large temperature gradients [17]. During the solidification process, hot tearing may occur due to the shrinkage of melt pools [18].

The surface roughness is greatly influenced by process parameters. The laser power, scanning speed, and their combined interaction (energy density) are often presented as the primary parameters affecting the surface roughness of LPBF-printed parts [19]. A high laser power tends to reduce the surface roughness, as the recoil pressure flattens out the melt pools and reduces the balling phenomenon by increasing wettability of melt pools. Low scanning speeds reduce top surface roughness but increase the side surface roughness [20]. The low hatch spacing ensures overlapping between melt tracks and leads to low surface roughness [21]. A small layer thickness ensures finer particle sizes and facilitates a more complete melting process due to the higher surface-to-volume ratio compared to a large layer thickness, which reduces the surface roughness [22]. Additionally, the energy density has a great influence on the surface roughness. A low energy density leads to discontinuous melt tracks caused by lacking wettability and formation of balling on melt tracks, resulting in poor surface roughness. A moderate energy density leads to low surface roughness because of the formation of continuous and smooth melt tracks with good wettability. However, an excessive energy density leads to the evaporation of powder and severe spattering, which seriously hampers the surface quality [23].

In conclusion, this work primarily investigated the influence of process parameters on the relative density, surface roughness, and mechanical properties of a Zr-4 alloy. Optimum parameters were determined to produce a near-fully dense Zr-4 alloy with low surface roughness. The microstructure, phase, and change of mechanical properties and surface roughness of Zr-4 alloy affected by process parameters are described, which provides guidance for the future research of Zr-4 alloy formation by LPBF.

## 2. Materials and Methods

### 2.1. Materials

**Figure 1:**
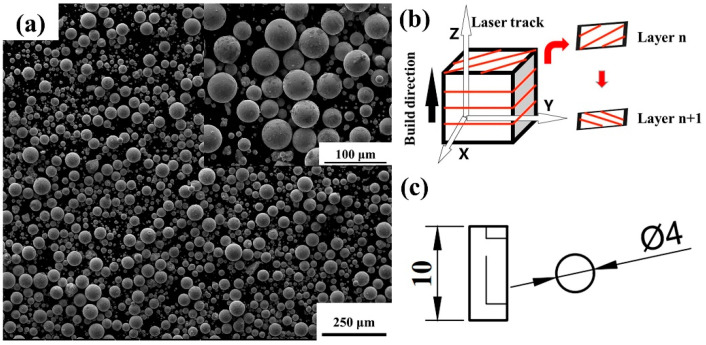
(**a**) Powder morphology of the Zr-4 alloy; (**b**) the schematic of the orthogonal scanning strategy; (**c**) geometries of the compressive sample model.

Spherical Zr-4 alloy powder (Hebei Baoju New Material Technology Co., LTD, Hebei province, China) prepared by the plasma method was used as the raw material, and the chemical composition of the powder is shown in Table 1. Figure 1a shows the morphology of the powder with a particle size ranging from 15 to 53 μm. The powder flowability was measured as 17.5 s/50 g using a Hall flow meter.

### 2.2. LPBF Process 

A Dimetal-100 LPBF equipment (Laseradd, Guangzhou, China) was employed to print Zr-4 alloy samples. The LPBF chamber was filled with argon atmosphere to ensure the oxygen content less than 0.01 vol%. The relative density of the Zr-4 alloy by LPBF is affected by the laser power, scanning speed, hatch spacing, and layer thickness [24,25]. Based on our previous studies on the parameter exploration, the laser power and scanning speed were in the range of 100–200 W and 400–1400 mm/s, respectively. The hatch spacing was fixed at 0.07 mm to ensure the adjacent melt tracks could overlap, and the layer thickness was fixed at 0.03 mm to enable every layer of the powder to be completely melted [19]. Compared with the fill line scan and the chess board scan, LPBF-printed samples can obtain lower porosity by the orthogonal scanning strategy [26], as shown in Figure 1b. Such a scanning strategy was thus adopted for printing. The specific process parameters are shown in Table 2.

### 2.3. Characterizations

Metallographic samples with dimensions of 10 × 10 × 10 mm^3^ were ground, polished and eventually etched by a corrosive solution (10 vol% HF, 45 vol% HNO_3_, and 45 vol% H_2_O) for 15 s [27,28]. A DMI-3000M optical microscope (OM, Leica Microsystems, Weztlar, Germany) and a FEI Quanta 250 scanning electron microscope (SEM, FEI, Portland, OR, USA) were used to observe the microstructure of the samples. The WDs of the SEM images ranged from 9 mm to 10 mm, and the voltage was set as 15 Kv. The phase composition was identified through X-ray diffraction (XRD, The X’Pert3 Powder diffractometer; Malvern Panalytical, Almelo, Holland). The printed samples were polished for the XRD characterization. The degree of 2 theta was from 30° to 100°, and the scan rate was 0.013°/min. The Archimedean drainage method was used to test the relative density, i.e., the ratio of the test density to the theoretical density (6.55 g/cm3). The Zr-4 material was insoluble in water, and thus the relative density of the Zr-4 alloy can be measured by the method. Three effective data were measured from the samples to determine the average relative density value. The top surface roughness of the Zr-4 alloy was measured by an ultra-depth of field microscope (VHX-5000, KEYENCE, Osaka, Japan). The compression test was carried out by an AG-IC 50kN electronic universal testing machine (SHIMADZU, Tokyo, Japan) with a compression rate of 0.2 mm/min. Three compressive samples for each set were used to estimate the compression properties according to the GB/T 7314-2017 standard. The models of Zr-4 samples for testing are shown in Figure 1c. The samples were printed without any support structures, which were removed from the baseplate by wire cutting. The fractures of the compressed samples were observed using SEM. The SPSS software (IBM, Almonk, NY, USA) was used to conduct the correlation analysis.

## 3. Results and Discussion

### 3.1. Influence of Laser Parameters on the Relative Density

**Figure 2:**
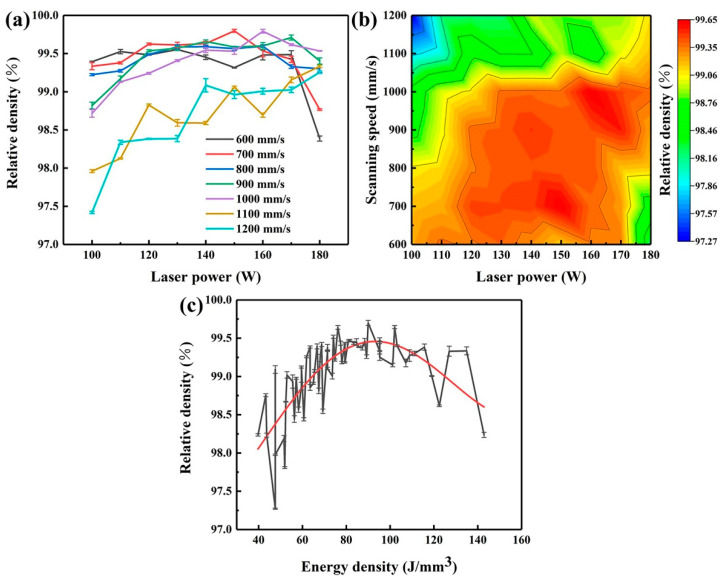
Effects of process parameters on the relative density of the Zr-4 alloy: (**a**) variation of relative density against the laser power and scanning speed; (**b**) iso-density diagram; (**c**) variation of the relative density against the energy density.

Figure 2a shows the variation of relative density of the LPBF-printed Zr-4 alloy with the laser power and scanning speed. When the scanning speed ranged from 600 mm/s to 1000 mm/s, the relative density of the alloy increased but then decreased with the increase in the laser power. When the scanning speed of 600 mm/s was applied, the relative density reached a peak of 99.40% ± 0.01% at the laser power of 130 W. At the scanning speed of 1000 mm/s, the highest relative density of 99.64% ± 0.02% could be achieved at the laser power of 160 W. The peak value of the relative density gradually increased and shifted to a higher scanning speed with the increase in the laser power. With the scanning speed of 1100 mm/s, the relative density of the alloy increased from 97.81% ± 0.02% to 99.18% ± 0.02%, as the laser power increased from 100 W to 180 W. Figure 2b exhibits the iso-density diagram of the LPBF-printed Zr-4 alloy with the laser power and scanning speed. When the laser power and scanning speed were in the ranges of 120–160 W and 600–1000 mm/s, respectively, the relative density of the alloy could remain larger than 99.3%, indicating an excellent densification. Noted that when the scanning speed exceeded 900 mm/s, the laser power should be increased to 170 W to ensure a high relative density.

The Archimedean drainage method has been widely used in the density measurement of LPBF-printed parts [29,30]. The LPBF-printed Zr-4 samples were fully dense with a relative density exceeding 99%. However, the limitation of the Archimedean drainage method is the accuracy to estimate true weight, as surface tension of liquids used in the method tends to increase the weight to affect the density [31].

The energy density is dominant to influence the relative density of the printed alloy, which can be described by:

(1) $$E=\frac{P}{VSH}$$

where *P* is the laser power (W), *V* is the scanning speed (mm/s), *S* is the hatch spacing (mm), and *H* is the layer thickness (mm). Figure 2c indicates the relationship between the relative density and the energy density. It can be concluded that the relative density tended to increase with the increasing energy density and deceased when the energy density exceeded 120 J/mm^3^. A high laser power and a low laser scanning speed can result in the accumulation of the laser energy in melt pools and the surge of the melt pool energy, causing excessive heat accumulation. Moreover, metallurgical defects such as cracks and pores could be induced. With the increase of the scanning speed, the energy of the melt pools gradually decreases. The melt pools become stable under a moderate laser power. A further increase in the scanning speed under a low laser power leads to insufficient energy within the melt pools, resulting in the increase of unmelted powder particles and pores, thus exhibiting a decreasing trend of the relative density [32,33,34]. As shown in Table 3, the Pearson correlation analysis was used to analyze the correlation between the energy density, the laser power, and the laser scanning speed via SPSS. The results showed the Pearson index between the laser power and the relative density was 0.244 while it was −0.517 between the laser scanning speed and the relative density, indicating the relative density of the LPBF-printed Zr-4 alloy was more sensitive to the laser scanning speed.

### 3.2. Influence of Laser Parameters on the Surface Roughness

**Figure 3:**
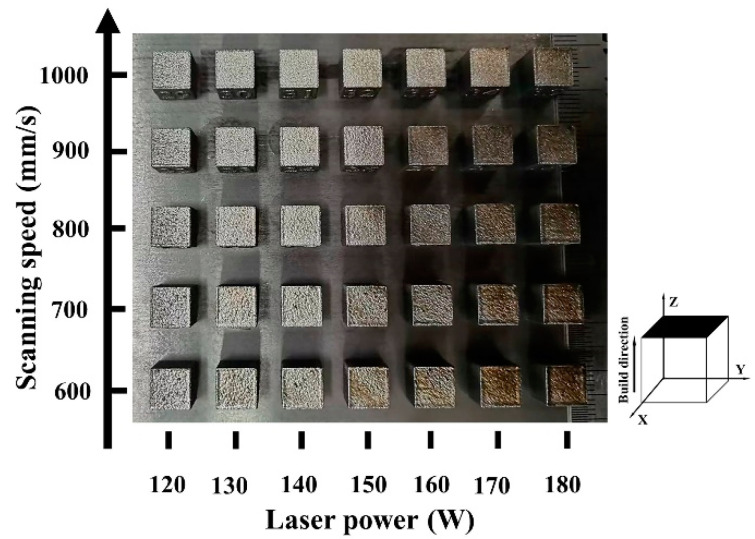
Macroscopic morphologies of the LPBF-printed Zr-4 samples with different process parameters.

The macroscopic morphologies of Zr-4 alloys by LPBF under different parameters are shown in Figure 3. A low laser power and a high scanning speed led to a smooth surface of the printed alloy. An overburn phenomenon occurred, when the laser power was greater than 150 W with a scanning speed of 600 mm/s. When the laser power was increased to 170 W, the printed alloy gradually obtained overburn surfaces at a scanning speed of 1000 mm/s. The generation of overburn surfaces can be attributed to the high laser energy density produced at high laser power and low scanning speed values [35,36]. 

Table 4 shows the surface roughness on the side for the samples printed with a laser power of 130 W and a scanning speed of 600–1200 mm/s. The results showed the influence of the scanning speed on the roughness of the side surface was not obvious. Generally, the side surface roughness is mainly affected by the layer thickness [19], which was fixed as 0.03 mm in this work. Additionally, the procedure of the contour scan with the same parameters used at each layer further reduced the distinction of the side surface roughness between different process parameters. 

**Figure 4:**
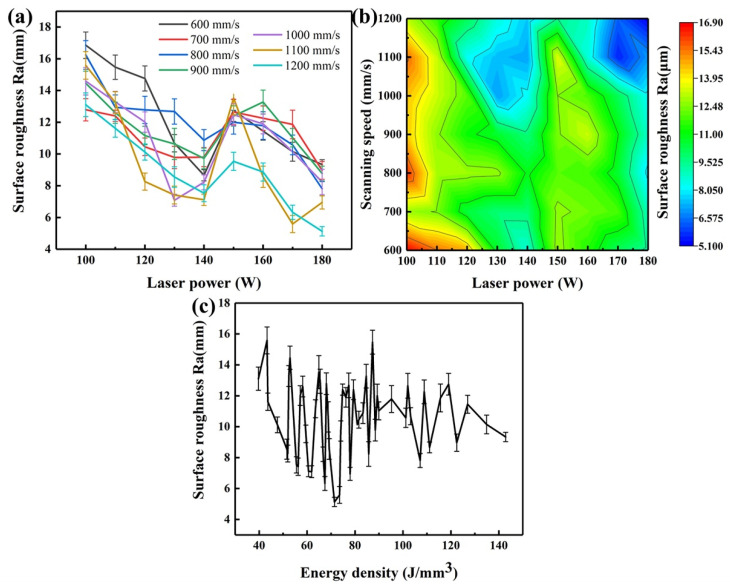
Effects of process parameters on the top surface roughness of the LPBF-printed Zr-4 alloy: (**a**) variation of the top surface roughness against the laser power and scanning speed; (**b**) iso-roughness diagram; (**c**) variation of the top surface roughness against the energy density.

As shown in Figure 4a, with the increase of the laser power at a certain scanning speed, the top surface roughness of the alloy tended to reduce. When the scanning speed of 1000 mm/s and the laser power of 100 W were applied, the maximum surface roughness of 14.58 ± 0.86 µm was obtained. The surface roughness decreased to the minimum value of 7.09 ± 0.38 µm, when the laser power increased to 130 W. As the laser power increased, the roughness began to rise to an Ra value of 12.47 ± 0.69 µm at 150 W. By further increasing the laser power to 180 W, the roughness value decreased to 8.24 ± 0.80 µm. Figure 4b shows the iso-roughness diagram of the top surface of the printed Zr-4 alloy. The low roughness of the alloy was achieved with a laser power of 120–140 W and a scanning speed of 1000–1100 mm/s. However, the laser power greater than 160 W and the scanning speed greater than 1100 mm/s also led to a low top surface roughness. In Figure 4c, the trend of the surface roughness against the energy density was similar with that of the laser power. A low surface roughness was obtained with the energy density in the range of 50–80 J/mm^3^ and exceeding 130 J/mm^3^.

**Figure 5:**
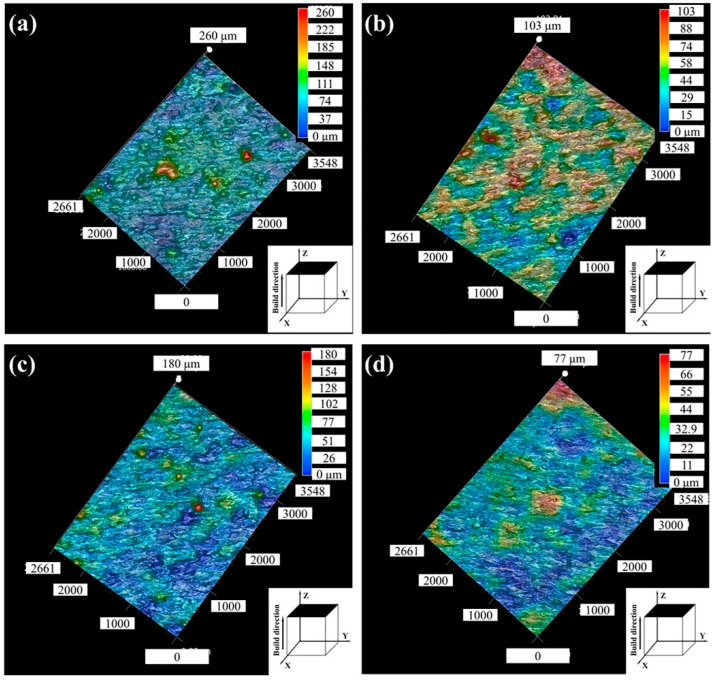
Top surface profiles under different laser powers (the scanning speed was fixed as 1100 mm/s): (**a**) *P* = 100 W; (**b**) *P* = 130 W; (**c**) *P* = 150 W; (**d**) *P* = 180 W.

Figure 5 shows the profiles of the top surface of the printed Zr-4 alloy with different laser power values. A lower laser power led to an insufficient laser input energy, and the Zr-4 powders could not be fully melted, resulting in the existence of bulges. Meanwhile, the viscosity of the low-energy melt pools increased; thus, the molten tracks became rugged and discontinuous [37]. Such factors led to the increase of the surface roughness (Figure 5a). With a further increase in the laser power to 140 W, the powder was completely melted, and the liquid molten pools were spread smoothly under the moderate power. Therefore, a low surface roughness of the alloy was obtained (Figure 5b). The uneven morphology was affected by the surface tension of liquid melt pools [38]. When the laser power increased to 160 W, it was easy to produce the periodizing effect and increase the surface protrusion due to the generation of the large temperature gradient between the center and the edge of the melt pools [36], resulting in the high surface roughness (Figure 5c). When the laser power was greater than 160 W, an obvious overburn phenomenon appeared on the surface [35], indicating that the laser energy was excessive. In this case, adjacent melt pools fused together before their solidification, and the lap between the melt pools was reduced. As a result, a giant bulge was created at the edge of the top surface (Figure 5d), which caused a low top surface roughness but warping deformation on all sides with a laser power of 180 W. Therefore, it can be concluded that the ideal surface roughness was obtained with the laser power in the range of 120–140 W.

### 3.3. Microstructure 

**Figure 6:**
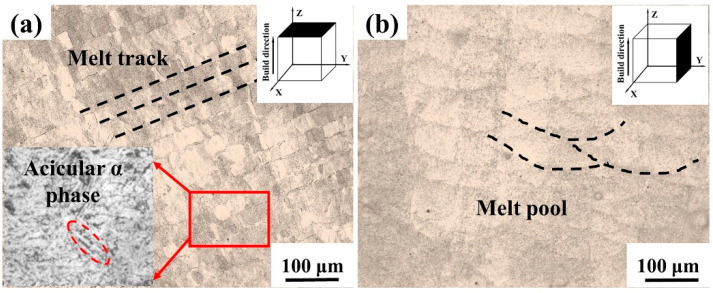
Metallographic images of the Zr-4 alloy printed using a laser power of 130 W and a scanning speed of 1000 mm/s along different planes: (**a**) the transverse plane; (**b**) the longitudinal plane.

Figure 6 shows the OM morphology of the LPBF-printed Zr-4 alloy with a laser power of 130 W and a scanning speed of 1000 mm/s. As observed in Figure 6a, the morphology of the alloy along the transverse plane exhibited acicular grains with a size of ~1 µm. Figure 6b exhibits overlapped molten pools without obvious metallurgical defects along the longitudinal plane. 

**Figure 7:**
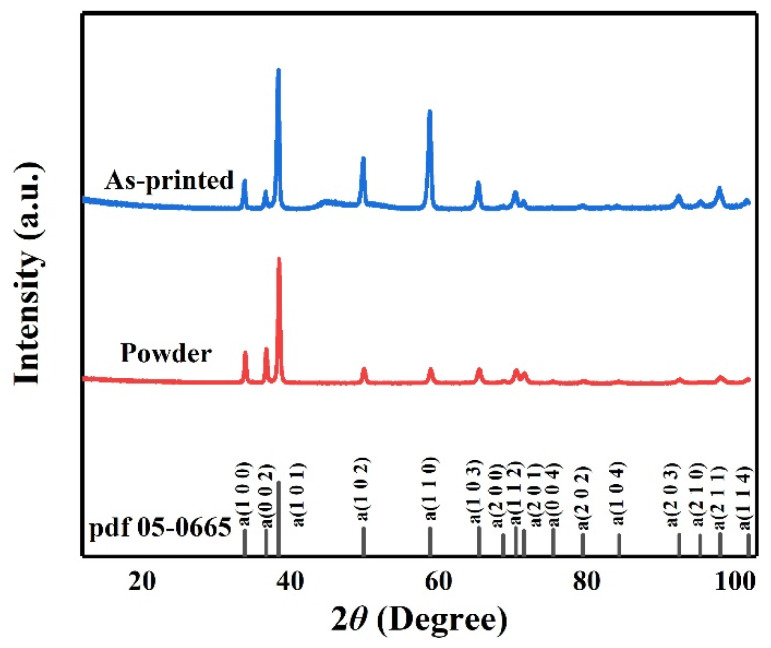
XRD pattern of the LPBF-printed Zr-4 alloy obtained using a laser power of 130 W and a scanning speed of 1000 mm/s.

Figure 7 shows the XRD diffraction pattern of the printed Zr-4 alloy with a laser power of 130 W and a scanning speed of 1000 mm/s. According to the standard diffraction pattern, an α-Zr phase could be identified in the printed Zr-4 alloy. During LPBF, the powder melted rapidly to form a liquid melt pool and solidified rapidly to form acicular martensite. In the rapid cooling stage, β-Zr, a stable phase at high temperatures in Zr alloys, undergoes the phase transition from β to α. Additionally, non-equilibrium phase transformation often occurs during the cooling process, producing metastable structures such as α'/α" phases. Because their crystal structures are similar to the α phase obtained by the equilibrium phase transformation, they cannot be separated in XRD patterns [39,40,41].

### 3.4. Compression Properties 

**Figure 8:**
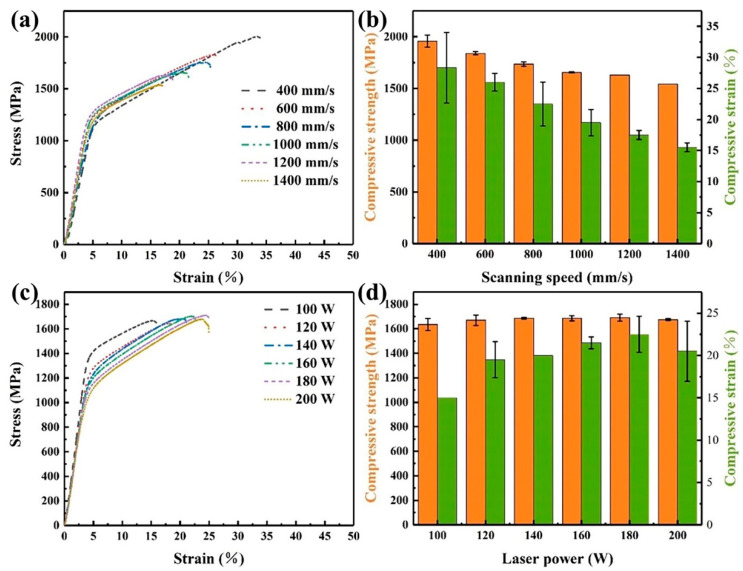
Compressive properties of the Zr-4 alloy at different laser powers and scanning speeds: (**a**) compressive curves at different scanning speeds; (**b**) variation of the compressive strength against the scanning speed (*P* = 130 W); (**c**) compressive curves at different laser powers; (**d**) variation of the compressive strength against the laser power (*V* = 1000 mm/s).

Our previous studies on the tensile properties showed the printed Zr-4 tensile coupons exhibited a quite low ductility. Even with the optimized parameters, the tensile coupons were still fractured at the very early stage of the testing. Therefore, the compression test was selected. Figure 8 shows the compressive properties of the LPBF-printed Zr-4 alloys with different parameters. When a laser power of 130 W was applied, both the compression strength and compression strain of the printed Zr-4 alloy decreased with the increase in the scanning speed (Figure 8a,b). The compressive strength gradually decreased from 1950 MPa with a scanning speed of 400 mm/s to 1540 MPa with a scanning speed of 1400 mm/s, while the compressive strain decreased from 28.3% to 15.5%. When the scanning speed was kept as 1000 mm/s, the compression strength of the printed alloy remained about 1650 MPa with an increase in the laser power from 100 W to 200 W (Figure 8c,d). When the laser power increased from 100 to 180W, the compression strain increased from 15% to 22.5% and then decreased to 20.5% as the laser power further increased to 200 W.

**Figure 9:**
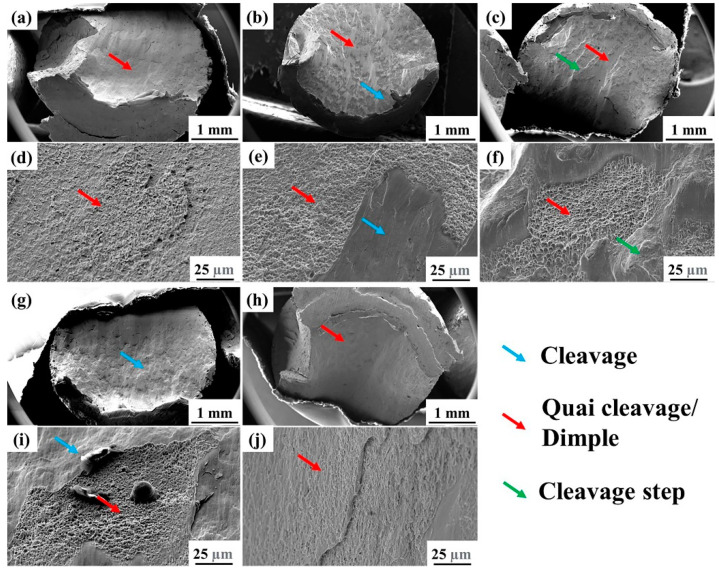
Compression fractures of the Zr-4 alloy under different laser parameters: (**a**,**d**) *P* = 130 W and *V* = 400 mm/s; (**b**,**e**) *P* = 130 W and *V* = 1000 mm/s; (**c**,**f**) *P* = 130 W and *V* = 1400 mm/s; (**g**,**i**) *P* = 100 W and *V* = 1000 mm/s; (**h**,**j**) *P* = 200 W and *V* = 1000 mm/s.

Figure 9 shows the compression fracture morphologies of the LPBF-printed Zr-4 alloys using different process parameters. The fracture surface of the printed alloy was mainly composed of the quasi-cleavage planes with a laser power of 130 W and a scanning speed of 400 mm/s (Figure 9a,d). When the scanning speed increased to 1000 mm/s, the fracture surface was composed of cleavage and quasi-cleavage planes (Figure 9b,e). When the scanning speed reached 1400 mm/s, obvious tearing edges and quasi-cleavage regions between them were found on the fracture surface (Figure 9c,f). With the increase in the scanning speed, the fracture surface gradually evolved from cleavage to semi-cleavage and semi-quasi-cleavage plane and finally became a dominated cleavage fracture. Such an increase in the cleavage region and a decrease in the quasi-cleavage and dimple regions are the main reasons for the deterioration of ductility [42].

Comparatively, as the scanning speed was fixed as 1000 mm/s, the fracture surface was relatively flat at the laser power of 100 W (Figure 9g). Cleavage planes could be observed with a few quasi-cleavage areas (Figure 9i). When the laser power increased to 130 W, the quasi-cleavage regions increased. When the laser power reached 200 W, the fracture surface presented an angle of 45° between the fracture surface and the horizontal plane (Figure 9h). Additionally, all the surfaces were presented as quasi-cleavage planes, where some possessed lamellar shedding boundaries (Figure 9j). The increase of the quasi-cleavage regions led to an increase in the compression strain with the increase of the laser power.

The changes in laser power and scanning speed led to the variation of the energy density. When a low laser power or high scanning speed was used, the resulted low laser energy density caused the formation of defects such as cracks and pores. For example, samples with a laser power of 130 W and a scanning speed of 1400 mm/s (energy density of 44 J/mm^3^) exhibited a relatively lower compressive strength (1540 MPa) and compressive strain (15%), corresponding to a low relative density of ~98.5% (Figure 2c). Therefore, with an increase in the laser power and a decrease in the scanning speed, the compressive strength of the printed alloy was improved. Additionally, the scanning speed played an important role in the interaction between the laser and the powder. A long interaction period tended to cause oxidation and nitriding, which limited the mobility of dislocation and hampered the ductility [43,44]. Consequently, a high energy density (80–120 J/mm^3^) and a low scanning speed (below 800 mm/s) were recommended during LPBF.

## 4. Conclusions

A Zr-4 alloy with a high relative density and excellent mechanical properties was manufactured by LPBF. Process optimization, surface roughness measurement, microstructure observation, and mechanical properties analysis were conducted. The main findings are presented as follows:

1. The LPBF process parameters for printing the Zr-4 alloy were optimized as the laser power of 120–160 W and the scanning speed of 600–1000 mm/s. A highest relative density greater than 99.3% could be obtained with the energy density of 70–110 J/mm^3^.
2. The top surface roughness of the alloy tended to reduce with the increase of the laser power as well as the energy density at a certain scanning speed. The low roughness of the alloy was achieved with laser powers of 120–140 W and scanning speeds of 1000–1100 mm/s and energy densities of 60–80 J/mm^3^.
3. The microstructure of the printed Zr-4 alloy consisted of an α-Zr phase with an average grain size of about 1 μm. The α-Zr existed in the form of crisscross acicular grains, determined by the temperature gradient of molten pools.
4. The laser power and scanning speed showed remarkable influences on the compressive strength and compressive strain of the Zr-4 alloys. Under the laser power of 130 W, the compressive strength gradually decreased from 1950 MPa to 1540 MPa and the compressive strain decreased from 28.3% to 15.5% with the increase in the scanning speed from 400 mm/s to 1400 mm/s. When the scanning speed was 1000 mm/s and the laser power increased from 100 W to 200 W, the compression strength remained about 1650 MPa while the compressive strain tended to increase from 15% to 22.5%. A high energy density (80–120 J/mm^3^) and a low scanning speed (below 800 mm/s) were recommended during LPBF.

## Figures and Tables

**Table 1:** Chemical composition of the Zr-4 powder.

Element	Cr	Sn	Fe	Zr
Wt. %	0.12	0.89	0.19	Bal.

**Table 2:** Parameters to print the Zr-4 alloy via LPBF.

Laser Power (W)	100, 110, 120, 130, 140, 150, 160, 170, 180, 190, 200
Scanning speed (mm/s)	400, 600, 700, 800, 900, 1000, 1200, and 1400
Hatch spacing (mm)	0.07
Layer thickness (mm)	0.03
Spot diameter (mm)	0.08
Scanning strategy	Orthogonal scanning

**Table 3:** Pearson correlation analysis between the energy density, the laser power, and the laser scanning speed.

		*P*	Relative Density			*V*	Relative Density
*P*	Pearson correlation	1	0.244	*V*	Pearson correlation	1	−0.517
	Significant		0.054		Significant		0
	number	63	63		number	63	63
Relative density	Pearson correlation	0.244	1	Relative density	Pearson correlation	−0.517	1
	Significant	0.054			Significant	0	
	number	63	63		number	63	63

**Table 4:** The side surface roughness of the Zr-4 alloy (the laser power was 130 W).

Scanning Speed (mm/s)	600	800	900	1000	1100	1200
Roughness (µm)	8.63 ± 0.55	9.25 ± 0.6	9.14 ± 1.57	9.89 ± 0.23	10.12 ± 0.59	9.49 ± 0.76

## Data Availability

Not applicable.
